# Risk factors for self-harm in people with epilepsy

**DOI:** 10.1007/s00415-018-9094-2

**Published:** 2018-10-24

**Authors:** Hayley C. Gorton, Roger T. Webb, W. Owen Pickrell, Matthew J. Carr, Darren M. Ashcroft

**Affiliations:** 1 Centre for Pharmacoepidemiology and Drug Safety, Division of Pharmacy and Optometry, School of Health Sciences, Faculty of Biology, Medicine and Health, University of Manchester, Manchester Academic Health Sciences Centre (MAHSC), 1.134 Stopford Building, Oxford Road, Manchester, M13 3PT UK; 2 NIHR Greater Manchester Patient Safety Translational Research Centre, University of Manchester, Manchester Academic Health Sciences Centre (MAHSC), Manchester, UK; 3 Centre for Mental Health and Safety, Division of Psychology and Mental Health, School of Health Sciences, Faculty of Biology, Medicine and Health, University of Manchester, Manchester Academic Health Sciences Centre (MAHSC), Manchester, UK; 4 Neurology and Molecular Neuroscience Research Group, Swansea University Medical School, Swansea University, Swansea, UK

**Keywords:** Epilepsy, Self-harm/self harm, Case–control, Cohort, Epidemiology

## Abstract

**Objective:**

To estimate the risk of self-harm in people with epilepsy and identify factors which influence this risk.

**Methods:**

We identified people with incident epilepsy in the Clinical Practice Research Datalink, linked to hospitalization and mortality data, in England (01/01/1998–03/31/2014). In Phase 1, we estimated risk of self-harm among people with epilepsy, versus those without, in a matched cohort study using a stratified Cox proportional hazards model. In Phase 2, we delineated a nested case–control study from the incident epilepsy cohort. People who had self-harmed (cases) were matched with up to 20 controls. From conditional logistic regression models, we estimated relative risk of self-harm associated with mental and physical illness comorbidity, contact with healthcare services and antiepileptic drug (AED) use.

**Results:**

Phase 1 included 11,690 people with epilepsy and 215,569 individuals without. We observed an adjusted hazard ratio of 5.31 (95% CI 4.08–6.89) for self-harm in the first year following epilepsy diagnosis and 3.31 (95% CI 2.85–3.84) in subsequent years. In Phase 2, there were 273 cases and 3790 controls. Elevated self-harm risk was associated with mental illness (OR 4.08, 95% CI 3.06–5.42), multiple general practitioner consultations, treatment with two AEDs versus monotherapy (OR 1.84, 95% CI 1.33–2.55) and AED treatment augmentation (OR 2.12, 95% CI 1.38–3.26).

**Conclusion:**

People with epilepsy have elevated self-harm risk, especially in the first year following diagnosis. Clinicians should adequately monitor these individuals and be especially vigilant to self-harm risk in people with epilepsy and comorbid mental illness, frequent healthcare service contact, those taking multiple AEDs and during treatment augmentation.

**Electronic supplementary material:**

The online version of this article (10.1007/s00415-018-9094-2) contains supplementary material, which is available to authorized users.

## Introduction

People with epilepsy are twice as likely to die by suicide compared to those without epilepsy [1]. Nonfatal self-harm, defined as any type of intentional self-injury or self-poisoning [2], may lie on the causal pathway between epilepsy and suicide. There are multiple motivations for engaging in self-harm, ranging from suicide attempt to emotional regulation without suicidal ideation [2]. Regardless of intent, self-harm is the strongest predictor of suicide [3].

Risk of hospitalization for self-harm in people with epilepsy has been estimated in two studies [4, 5]. Singhal et al. reported a relative risk of 3.9 (95% CI 3.8–4.1) for self-harm in the year following hospitalization for epilepsy and 2.6 (95% CI 2.5–2.7) in subsequent years [4]. Meyer et al. estimated the hospital self-harm presentation rate in people with epilepsy to be 2.04 (95% CI 1.85–2.25) times that of the comparison group [5]. Meyer et al. identified epilepsy diagnosis from the self-harm reporting form, as part of a multi-centre study, and confirmed with review of medical notes [5]. Singhal et al. identified people with epilepsy from recorded hospital admission or day case contact due to epilepsy [4]. It is possible, therefore, that this may have included only individuals with the most severe or poorly managed epilepsy, which resulted in hospital presentation. Both studies required individuals to be hospitalized for the self-harm event, thus do not include those who presented in the community for self-harm. A previous study conducted in a UK primary care dataset estimated an odds ratio for self-harm of (2.35, 95% CI 1.67–3.29) for people with epilepsy compared to those without [6]. Self-harm cases were defined from those reported in primary care only, as this study was conducted before it was possible to link this dataset with hospital records. It is not known whether this magnitude of increased risk for self-harm is observed in a primary care patient cohort when linked to hospital reports of self-harm and national mortality records.

The World Health Organization (WHO) recommends that people with epilepsy should be asked about self-harming thoughts and behaviours in certain, specific circumstances [7]. However, there may be additional factors that could alert clinicians to instigate this discussion. To our knowledge, the factors that influence someone with epilepsy to self-harm have not been identified.

We, therefore, aimed to: (1) estimate self-harm risk in persons with epilepsy versus those without; and (2) identify risk factors for self-harm among individuals with epilepsy.

## Methods

### Setting

We extracted an incident epilepsy cohort from the Clinical Practice Research Datalink (CPRD), linked to Hospital Episode Statistics (HES) and Office for National Statistics (ONS) mortality data. The CPRD is a primary care dataset that contains routinely collected electronic health records capturing information on patient demographics, diagnoses and treatments in general practice. It has been shown to be representative of the UK population [8]. All of the linked general practices were located in England, representing 75% of all English practices included in the CPRD at the time of data extraction. We used the linked subset of the July 2015 version containing 7,378,852 individuals from 378 general practices with data deemed to be of sufficient quality for conducting research. HES contains hospital inpatient discharge dates and diagnoses, and ONS mortality data include date and cause of death.

The study was approved by the Independent Scientific Advisory Committee (protocol 17_063R) of the CPRD. Informed consent is not required for studies that use anonymized data from the CPRD.

### Study population: incident epilepsy cohort

From the CPRD, we extracted the incident epilepsy cohort that formed the basis for both Phase 1 and Phase 2. The study observation period was 01/01/1998–31/03/2014 to correspond with linkage availability. We used our previously published definition to identify people with epilepsy [1] which requires a diagnostic code for epilepsy and an associated prescription for an antiepileptic drug (AED) [9, 10]. We defined the epilepsy index date as the latest of the epilepsy diagnosis date and AED prescription in the 6 months prior, or 1 month after diagnosis. We restricted to the incident epilepsy cohort by mandating at least 12 months registration prior to epilepsy index date, and no prior epilepsy diagnosis in this look-back period. This minimized the risk of ‘prevalent-user bias’, whereby the timing of epilepsy onset could confound the relationship with self-harm risk [11]. We required individuals to be without history of self-harm in the look-back. We restricted the cohort to persons aged ten or older, as this is the minimum age at which the WHO recommend that clinicians should discuss self-harm [7]. This threshold also aligns with previously published studies, because self-harm intent is particularly difficult to discern below age ten [12].

### Phase 1: matched cohort study

We matched each person with incident epilepsy to up to 20 individuals without epilepsy on gender, year of birth (± 2 years) and general practice. Individuals sampled for the comparison cohort had not received a diagnostic code for epilepsy or self-harm in the look-back period, and had been registered for at least 12 months at the practice. Individuals were followed up until the earliest date of: first self-harm event, death, patient transferred out of practice, latest date of data collection from the practice, or end of the study’s observation period.

### Phase 2: nested case–control study

From the cohort of people with incident epilepsy, we identified first recorded cases of self-harm during the study window—the self-harm case date. We matched these cases to up to 20 control individuals from within the incident epilepsy cohort, without history of self-harm on the self-harm case date, using incidence-density sampling [13]. We matched cases to controls on gender, year of birth (± 2 years) and timing of incident epilepsy diagnosis (± 1 year), because these variables may confound the relationship between the exposures investigated and self-harm risk [14].

## Outcomes

We included both fatal (suicide) and nonfatal self-harm in our definition. We identified self-harm from primary care records using clinician-verified Read codes [15] and from HES using the following ICD-10 codes: X60-84, Y87.0 and Y87.2. We used these same codes to identify suicide from ONS mortality data, with the addition of Y10-34 (excluding Y33.9) [16]. These codes represent undetermined intent, which is included in the ONS definition of suicide [17]. As this conclusion is assigned by a coroner in the UK, it is not appropriate to apply the same codes to nonfatal self-harm.

In Phase 2, we investigated multiple exposures. We identified level of deprivation by quintiles of Index of Multiple Deprivation (IMD-2010), and compared to the least deprived quintile (1st quintile). We identified mental illness diagnoses (alcohol misuse, anxiety disorder, bipolar disorder, depression, eating disorder, personality disorder and schizophrenia) from primary care data and HES using previously published codes [15, 18] that were recorded prior to self-harm case date. We developed a code list for substance misuse that was independently verified by two general practitioners (GPs) and is available at http://www.clinicalcodes.org [19]. We identified referrals to psychiatric services in the year prior to self-harm case date from Family Health Services Authority and National Health Service speciality fields in the CPRD [20]. Contact with healthcare services was measured by number of face-to-face consultations with the GP and number of hospitalizations for any reason, in the year prior to self-harm case date. Physical illness comorbidity was measured by assignment of a Charlson index score using Read codes from the CPRD [21] and ICD-10 codes from HES. The Charlson index is a measure of comorbidity, based on 1-year mortality risk derived from 17 comorbidities [21]. We measured AED utilization in two ways. First, we counted the number of AED types that the person was exposed to in the 90 days prior to self-harm case date and compared this to AED monotherapy. Second, we determined if there had been augmentation of AED treatment in the 6 months prior to self-harm index data. Due to the recommendation of slow withdrawal of AEDs when changing therapy [22], we defined augmentation as persistence of two AEDs 90 days after the introduction of the additional AED.

### Statistical analysis

In Phase 1, we estimated the relative risk of self-harm in the incident epilepsy versus comparison cohorts using a stratified Cox proportional hazards model. We adjusted for level of deprivation because both epilepsy [23] and self-harm [15] are independently associated with higher levels of deprivation, which may confound any observed associations. We assessed the proportionality assumption using a formal test that compared Schoenfeld residuals, with a *p* value < 0.05 indicating non-proportionality [24], and by graphical inspection. We reported baseline characteristics as numerical and percentage frequencies and medians, and estimated prevalence ratios for pre-existing mental illness diagnoses and types of prescribed psychotropic medication. In Phase 2, we used conditional logistic regression to estimated exposure odds ratios to indicate relative risk of self-harm associated with the following exposures: (1) level of deprivation; (2) mental illness; (3) referral to psychiatric services; (4) contact with healthcare services; (5) physical illness comorbidity; and (6) AED utilization. Data analysis for both phases was undertaken using Stata, version 13 (StataCorp, College Station, TX, USA).

## Results

### Phase 1: matched cohort study

We matched 11,690 people with incident epilepsy (median age 53, IQR 30–72; 52% male) to 215,569 persons without epilepsy. Compared to the matched cohort, the epilepsy cohort was more deprived and more likely to have been diagnosed with any mental illness, treated with psychotropic medication or opioids (Table 1). The median follow-up times were 3.6 years (IQR 1.3–7.2) and 4.7 years (IQR 2.0–8.3) for the epilepsy and comparison cohorts, respectively.

**Table 1:** Prevalence of diagnosed mental illnesses and psychotropic medication prescribing at baseline

Baseline characteristics	Epilepsy cohort (*n* = 11,690)	Comparison cohort (*n* = 215,569)	Prevalence ratios (95% CI)
Previous diagnoses			
Alcohol misuse^a^	596 (5.1%)	2433 (1.1%)	4.53 (4.14–4.95)
Anxiety disorders^a^	2053 (17.6%)	26,405 (12.3%)	1.43 (1.37–1.50)
Bipolar disorder^a^	87 (0.7%)	718 (0.3%)	2.25 (1.80–2.81)
Depression^a^	2475 (21.2%)	30,084 (14.0%)	1.52 (1.45–1.58)
Eating disorders^a^	138 (1.2%)	1337 (0.6%)	1.88 (1.58–2.24)
Personality disorders^a^	83 (0.7%)	567 (0.3%)	2.72 (2.16–3.42)
Schizophrenia-spectrum disorders^a^	238 (2.0%)	1420 (0.7%)	3.09 (2.69–3.55)
Substance misuse^a^	660 (5.7%)	2316 (1.1%)	5.21 (4.77–5.68)
Prior prescription			
Antidepressant^a^	3952 (33.8%)	46,714 (21.7%)	1.56 (1.51–1.61)
Antipsychotic^a^	2318 (19.8%)	27,165 (12.6%)	1.59 (1.52–1.66)
Anxiolytic/hypnotic^a^	3527 (30.1%)	36,609 (17.0%)	1.50 (1.04–2.02)
Lithium^a^	38 (0.3%)	491 (0.2%)	1.79 (1.73–1.85)
Opioid^a^	5137 (43.9%)	76,516 (35.5%)	1.25 (1.21–1.28)

^a^ *p* < 0.05

There were 273 first self-harm events in the epilepsy cohort and 1547 in the comparison cohort. The overall incidence rates for first self-harm event (Table 2) were greater in the epilepsy cohort (5.0 per 1000 person-years, 95% CI 4.4–5.6) than in the comparison cohort (1.3 per 1000 person-years, 95% CI 1.3–1.4). The proportionality assumption for the stratified Cox proportional hazards model did not hold (*p* = 0.007); therefore, we divided follow-up time to first year after diagnosis and subsequent years. There was an excess risk of self-harm during the first year of follow-up (deprivation-adjusted HR 5.31, 95% CI 4.08–6.89) compared to subsequent years (deprivation-adjusted HR 3.31, 95% CI 2.85–3.84), although elevated risk persisted throughout the follow-up period.

**Table 2:** Incident rates and relative risks of first self-harm event

First self-harm	Epilepsy (n = 11,690, PY = 54,692)		Comparison cohort (n = 215,569, PY = 1,170,253)		Unadjusted HR (95% CI)	Deprivation-adjusted HR (95% CI)
	Number	Incidence/1000 PYs (95% CI)	Number	Incidence/1000 PYs (95% CI)		
Any time during follow-up	273	5.0 (4.4–5.6)	1547	1.3 (1.3–1.4)	3.82 (3.36–4.34)	3.67 (3.22–4.17)
0–1 year of follow-up	72	6.9 (5.4–8.7)	253	1.3 (1.1–1.4)	5.50 (4.24–7.15)	5.31 (4.08–6.89)
After 1 year of follow-up	201	4.5 (3.9–5.2)	1294	1.3 (1.3–1.4)	3.45 (2.97-4.00)	3.31 (2.85–3.84)

Number of self-harm events and incidence rates of self-harm per 1000 person-years, in epilepsy and comparison cohorts. Unadjusted hazard ratios and hazard ratios adjusted for level of deprivation indicate relative risk of self-harm

### Phase 2: nested case–control study

Within the epilepsy cohort we identified 273 individuals with a first self-harm event (cases) and matched them to 3790 control patients with epilepsy and without history of self-harm on the self-harm case date (Table 3). The median age was 34 years (IQR 20–46) and 43% were male. The median time since epilepsy diagnosis was 2.6 years (IQR 0.9–4.6) for persons who had self-harmed and 2.2 years (IQR 1.0–3.9) for control patients. Individuals living in the most deprived areas had an elevated self-harm risk compared to those living in the least deprived localities (5th quintile: OR 2.22, 95% CI 1.44–3.42, 4th quintile: OR 1.75, 95%CI 1.11–2.75), but there was no evidence of increased risk associated with other quintiles of deprivation.

**Table 3:** Exposure odds ratios indicating risk factors for self-harm in the epilepsy cohort

Risk factors	Cases (*n* = 273)	Controls (*n* = 3790)	Odds ratio (95% CI)
Level of deprivation			
1 (least deprived)	30 (11.0%)	689 (18.2%)	1 (ref)
2	51 (18.7%)	804 (21.2%)	1.43 (0.90–2.28)
3	47 (17.2%)	653 (17.2%)	1.61 (1.00-2.59)
4	59 (21.6%)	765 (20.2%)	1.75 (1.11–2.75)
5	86 (31.5%)	869 (22.9%)	2.22 (1.44–3.42)
missing	0	10 (0.3%)	N/A
Any mental illness diagnosis	180 (65.9%)	1350 (35.6%)	4.08 (3.06–5.42)
Number of mental illness diagnoses			
0	93 (34.1%)	2440 (64.4%)	1 (ref)
1	81 (29.7%)	764 (20.2%)	3.24 (2.34–4.50)
2	40 (14.6%)	445 (11.7%)	3.07 (2.03–4.66)
3 or more	59 (21.6%)	141 (3.7%)	15.36 (10.03–23.51)
Prior history of diagnoses			
Alcohol misuse	54 (19.8%)	186 (4.9%)	5.31 (3.70–7.94)
Anxiety disorders	97 (35.5%)	763 (20.1%)	2.28 (1.73–3.01)
Bipolar disorder	9 (3.3%)	27 (0.7%)	4.02 (1.87–8.64)
Depression	130 (47.6%)	812 (21.4%)	3.92 (2.94–5.22)
Eating disorders	12 (4.4%)	56 (1.5%)	3.22 (1.69–6.13)
Personality disorders	11 (4.0%)	19 (0.5%)	8.32 (3.83-18.0)
Schizophrenia-spectrum disorders	15 (5.5%)	74 (2.0%)	2.77 (1.56–4.95)
Substance misuse	57 (20.9%)	207 (5.5%)	5.17 (3.61–7.41)
Charlson comorbidity index			
0	151 (55.3%)	2267 (59.8%)	1 (ref)
1	66 (24.2%)	950 (25.1%)	1.07 (0.79–1.45)
2 or 3	31 (11.4%)	425 (11.2%)	1.14 (0.75–1.74)
4 or more	25 (9.1%)	148 (3.9%)	2.91 (1.75–4.82)

Mental illness diagnoses and diagnoses included in the Charlson comorbidity index were included if ever recorded prior to self-harm case date

There was no difference in self-harm risk associated with a Charlson comorbidity index score of 1 or 2–3, but an increased risk was evident when the score was 4 or more (OR 2.91, 95% CI 1.75–4.82). 65.9% of cases and 35.6% of controls had a history of mental illness. Having one or more mental illness diagnoses increased self-harm risk compared to having no such diagnoses (OR 4.08, 95% CI 3.06–5.42) and this risk increased markedly among individuals who had received three or more mental illness diagnoses (OR 15.36, 95% CI 10.03–23.51).

All mental illnesses examined were associated with an increased self-harm risk, but the magnitude varied across the diagnostic categories. Depression was the most common diagnosis and was associated with approximately a fourfold elevation in self-harm risk (OR 3.92, 95% CI 2.94–5.22). In a post hoc sensitivity analysis, we included depression symptom codes as well as diagnoses in the definition of depression. This did not alter the estimated risk (OR 4.03, 95%CI 3.04–5.33).

In the 12 months prior to self-harm case date, 12.8% of cases and 3.8% of controls were referred to specialist psychiatric services (OR 3.65, 95% CI 2.45–5.44). In the same timeframe, 45.8% of self-harm cases and 29.1% of controls were hospitalized at least once for any reason (OR 2.12, 95% CI 1.64–2.76). The median number of face-to-face consultations with a GP in the 12 months preceding the self-harm case date was nine (IQR 5–15) for cases and six (IQR 3–11) for controls. Compared to individuals who had 0–4 consultations in the previous year, individuals who had five or more consultations were at a two- to fivefold increased self-harm risk.

In the 90 days prior to self-harm case date, compared to individuals who were prescribed a single AED, those prescribed no AED (OR 1.47, 95% CI 1.01–2.12), two (OR 1.84, 95% CI 1.33–2.55) or three or more AEDs (OR 2.44, 95% CI 1.51–3.94) were at an increased risk of self-harm (Table 4). Augmentation of AED treatment in the prior 6 months was associated with a twofold increased risk of self-harm compared to no augmentation (OR 2.12, 95% CI 1.38–3.26).

**Table 4:** AED utilization in cases and controls

AED utilization	Cases (*n* = 273)	Controls (*n* = 3790)	OR (95% CI)
Current number of AEDs			
0	43 (15.8%)	486 (12.8%)	1.47 (1.01–2.12)
1	152 (55.6%)	2644 (69.8%)	1 (ref)
2	56 (20.5%)	507 (13.4%)	1.84 (1.33–2.55)
3 or more	22 (8.1%)	153 (4.0%)	2.44 (1.51–3.94)
Augmentation in prior 6 months			
0	202 (87.8%)	3588 (94.7%)	1 (ref)
1 or more	28 (12.2%)	202 (5.3%)	2.12 (1.38–3.26)

Number of types of AEDs prescribed in the 90 days preceding self-harm case date and evidence of treatment augmentation in the 6 months prior to self-harm case date

## Discussion

In a large population-based cohort study, we found that people with epilepsy have an elevated self-harm risk compared to those without the condition. There was a fivefold elevation in risk in the first year following diagnosis and a threefold increased risk persisting beyond this first year. Among people with epilepsy, those most likely to self-harm included people with comorbid mental illness diagnoses, previous psychiatric referral, previous hospitalization for any reason, or five or more consultations with their GP in the previous year. Individuals treated with none or multiple AEDs, including those who had recently augmented treatment, were at increased risk of self-harm, compared to those prescribed AED monotherapy.

We report the first published estimates for elevated self-harm risk in people with incident epilepsy in which self-harm cases were ascertained using both primary and secondary care records. Our estimates are slightly higher than those reported in earlier studies that included only individuals who presented to hospital with self-harm [4, 5] and estimates from the study using the predecessor to the CPRD, prior to linkage availability (OR 2.35, 95% CI 1.67–3.29), thus including only self-harm episodes that were recorded in primary care [6]. Previous studies did not restrict to an incident epilepsy cohort; therefore, inclusion of individuals with prevalent epilepsy may have resulted in prevalent-user bias [11]. This may have diluted the period of highest risk, close to the time of incident epilepsy diagnosis.

Among people with epilepsy, we found that elevated self-harm risk was associated with prior diagnosis of any mental illness or referral to psychiatric services. This corroborates with evidence reported from general population studies in which mental illness is associated with a 6- to 14-fold increased risk of self-harm, dependent on the specific diagnosis [4]. Within the epilepsy cohort, a fivefold increased risk of self-harm was associated with history of alcohol and substance misuse. It is possible that these individuals experience a high frequency of seizures, caused by the alcohol or substance misuse, or due to non-compliance with treatment as a result of a disordered lifestyle. This could contribute to the increased self-harm risk experienced by these individuals. A bidirectional relationship between attempted suicide, which includes self-harm, and epilepsy has been suggested previously [25].

Having five or more face-to-face general practice consultations in the previous year was associated with elevated self-harm risk, compared to people who attend up to four times per year. Clinicians should be alert to the risk of self-harm in individuals who present regularly, which may be in relation to epilepsy severity or comorbid conditions. Importantly, clinicians can use these frequent interactions to discuss self-harm risk with patients in this group.

The use of multiple AEDs is a result of treatment augmentation, due to inadequate seizure control; or during a period of switching to an alternative monotherapy due to lack of tolerance or for other reasons such as pregnancy [22]. The elevated self-harm risk observed during use of multiple AEDs is likely to be an indication of more severe epilepsy with an associated higher seizure frequency, which is not controlled by AED monotherapy. Furthermore, individuals who have many seizures may experience consequent psychosocial difficulties, including inability to drive or absence from work or social activities, which may exacerbate the stigma associated with epilepsy [26]. Additionally, some individuals may become despondent if AED treatment requires augmentation, despite compliance with monotherapy. This may result in difficulty coping and the condition may be perceived as a burden to the individual, both of which are known motivators of suicidal behaviour [27]. Indeed, we observed an elevated risk of self-harm associated with recent augmentation of AED treatment. We have previously identified the need to examine the risk associated with individual AEDs using carefully designed, new-user studies [28]. This was not the aim of this study; therefore, the study design does not allow us to comment on individual AEDs.

Self-harm risk was also elevated for people who were not prescribed an AED in the 90 days prior to the index self-harm case date (OR 1.47, 95% CI 1.01–2.12). On entry to the incident epilepsy cohort, all individuals were prescribed an AED. Therefore, those individuals without AED prescription on the self-harm case date may have gradually stopped taking AEDs because they became seizure free. In the UK, the National Institute for Health and Care Excellence (NICE) recommends that AED withdrawal should only be considered following a 2-year absence of seizures [21]. Given that the median time since epilepsy diagnosis on self-harm case date was approximately 2 years, it is unlikely that all of those who had no recent AED prescription withdrew their AED on the advice of a clinician. It is possible that some of those individuals were non-compliant with their medication regimen. This may be motivated by undesirable adverse events, beliefs about medication and illness, comorbid mental illness or lifestyle choices, all of which may potentially contribute to elevated self-harm risk.

Healthcare professionals involved in the care of people with epilepsy could instigate conversations about self-harm risk, especially if the described risk factors are present. These include mental health problems, and extend to both the clinicians responsible for the mental health services and those working in general primary care settings. Furthermore, GPs should consider discussing self-harm risk management in people who consult frequently. Further research could investigate whether any technological prompts could aid this during consultations.

### Strengths and limitations

This is the first published study to estimate self-harm risk among people with epilepsy in a large, linked primary care patient cohort, including 11,690 people with incident epilepsy. Linkage to HES maximized self-harm case ascertainment. The Read codes used to identify self-harm cases were verified by clinicians and have been used in other studies [15, 29]. To mitigate confounding by previous self-harm, we restricted the incident epilepsy cohort to include only those persons with no prior recorded history of self-harm in either their primary or secondary healthcare records. It is still possible, however, that individuals had a self-harm event prior to this look-back period and before their CPRD records began. Furthermore, we recognize that not all people who have a self-harm episode will present to healthcare services and those who do represent the “tip of the iceberg” of self-harm events [30]. However, our inclusion of self-harm reported to both primary and secondary care builds upon those studies which used only one of those sources to ascertain self-harm [4–6]. As people with epilepsy attend the GP more often than those who do not, there may have been more opportunity to report self-harm and they may be asked about self-harm as per the WHO recommendations [7]. This would overestimate the magnitude of elevated self-harm risk in people with epilepsy compared to those without the condition.

It is not possible to accurately determine the type of epilepsy from UK general practice data; therefore, this is something we could not examine in this study. Epilepsy type may influence risk of self-harm [31]. It would, therefore, be beneficial to compare self-harm risk among people with different epilepsy subtypes, particularly whether having symptomatic epilepsy (and therefore underlying brain pathology) has an influence on self-harm risk.

## Conclusion

In conclusion, clinicians should be aware that people with epilepsy are at increased risk of self-harm, compared to those without the condition, especially during the first year post-diagnosis. These patients should, therefore, be routinely monitored. Additionally, we recommend that clinicians are particularly vigilant for self-harm thoughts and behaviours in people with epilepsy and comorbid mental illness, those who consult regularly, those prescribed AED polytherapy and during periods of AED treatment augmentation.

## Electronic supplementary material

Below is the link to the electronic supplementary material.

Supplementary material 1 (DOCX 20 KB)
